# Dynamics of COVID-19 transmission including indirect transmission mechanisms: a mathematical analysis

**DOI:** 10.1017/S0950268820002563

**Published:** 2020-10-23

**Authors:** A. Meiksin

**Affiliations:** School of Physics and Astronomy, University of Edinburgh, James Clerk Maxwell Building, Peter Guthrie Tait Road, Edinburgh EH9 3FD, UK

**Keywords:** COVID-19, infectious disease epidemiology, mathematical modelling

## Abstract

The outbreak of the novel coronavirus severe acute respiratory syndrome-coronavirus-2 has raised major health policy questions and dilemmas. Whilst respiratory droplets are believed to be the dominant transmission mechanisms, indirect transmission may also occur through shared contact of contaminated common objects that is not directly curtailed by a lockdown. The conditions under which contaminated common objects may lead to significant spread of coronavirus disease 2019 during lockdown and its easing is examined using the susceptible-exposed-infectious-removed model with a fomite term added. Modelling the weekly death rate in the UK, a maximum-likelihood analysis finds a statistically significant fomite contribution, with 0.009 ± 0.001 (95% CI) infection-inducing fomites introduced into the environment per day per infectious person. Post-lockdown, comparison with the prediction of a corresponding counterfactual model with no fomite transmission suggests fomites, through enhancing the overall transmission rate, may have contributed to as much as 25% of the deaths following lockdown. It is suggested that adding a fomite term to more complex simulations may assist in the understanding of the spread of the illness and in making policy decisions to control it.

## Introduction

On 23 March 2020, the UK government introduced a partial lockdown in an attempt to curtail the spread of coronavirus disease-2019 (COVID-19) through the transmission of severe acute respiratory syndrome-coronavirus-2. Leaving home was allowed only for essential reasons: food, health and work. Just over three weeks after the partial lockdown, the weekly death rate of registered COVID-19 deaths peaked at 9495 [1], but had fallen to 6680 two weeks later, and continued to decline through July. Allowing for the time from exposure to death, the decline is evidence that non-pharmaceutical intervention successfully suppressed the spread of the epidemic [2, 3].

The main transmission mechanisms of COVID-19 are believed to be through viral-loaded respiratory droplets and close contact [4], although fomites [4, 5] and respiratory aerosols [4, 5, 6] are also suspected to be factors in the transmission. The restrictions on movement, whilst reducing person-to-person direct transmission, potentially continued to allow transmission through the indirect means of objects contaminated by an infectious person. Although viable amounts of the SARS-CoV-2 virus survive under laboratory conditions on contaminated surfaces [5] and articles in proximity to an infectious patient may show traces of the viral RNA [7], it has not been demonstrated that viable viruses survive in a natural environment in sufficient concentration to transmit the infection through this route. On the other hand, experiments suggest the lifetime of SARS-CoV-2 on fomites is prolonged in a protein-rich environment like airway secretions [8].

The relative importance of indirect transmission compared with direct is unknown, even under lockdown conditions. The World Health Organization (WHO) reports there is no conclusive evidence for fomite transmission, direct evidence for which is complicated by the frequent presence of infectious individuals with the fomites, making it difficult to establish which is the causative agent [4]. The report none the less cautions that the consistent presence of fomites in the environment of infected cases suggests fomite transmission is an active means of transmission of the SARS-CoV-2 virus, as it is for other coronaviruses.

Epidemic stochastic models and simulations (e.g. [3, 9, 10, 11]), generally do not include transmission by fomites, as the effective reproduction number may be adjusted for their effects to account for gross population statistics such as infection and death rates. As discussed below, direct estimates of the rate of fomite transmission are made difficult by the rarity of fomites in the general population. Yet the policy implications for transmission through direct and indirect transmissions may differ. Given that a moderately high proportion of the infectious population is suspected to be asymptomatic [4], there is a potential for infectious individuals working in essential services and who have not yet had reason to self-isolate, to unwittingly contaminate material that reaches the public with respiratory droplets. Whilst a lockdown will curtail direct transmission, indirect communication of the virus through essential services such as post deliveries or food supplies may be relatively unaffected. Additional policies may be required to mitigate their effects.

As an alternative to direct case studies for establishing the prevalence of fomite transmission of COVID-19, this note seeks to constrain the possible impact of indirect transmission through population modelling using the SEIR model with an added fomite term. As discussed in the next section, the constraint is nearly independent of the nature of the fomites, depending only weakly on the decay times of viruses on fomites. To focus the analysis, transmission within the UK is examined. An illustrative example is also presented of the possible implications for postal deliveries in the UK, although only upper limits may be determined for any particular source of fomite transmission since they all add together to the net fomite contribution inferred from a global population analysis.

## Methods

### Model equations

The standard set of SEIR differential equations for a population follows the dynamics of four sub-populations: the fraction *s* of the population susceptible to infection, the fraction *e* exposed to infection, the fraction *i* of infectious individuals and the fraction *r* of removed or recovered individuals. It is assumed no removed individual becomes susceptible again. Sub-populations *s* and *i* are coupled through a term *R_t_si/D_i_* where *R_t_*, the (time-dependent) effective reproduction number, is the average number of people an infectious person infects. The exposed and infectious periods are assumed to be exponentially distributed in time, with mean durations *D_e_* and *D_i_*, respectively.

A fomite term *f* is added to represent the number of contaminated objects per capita. If *C_f_* is the average number of potentially contaminated objects a person comes into contact with per day, then *C_f_i* is the per capita number of objects contaminated per day. (The infectious fraction among individuals able to contaminate the objects is assumed the same as in the general population.) The possibility of inter-article contamination is not included. It is assumed a contaminated object transmits the infection to an average *T_f_* members of the susceptible population. The coupling term between the susceptible population and fomites is then *T_f_s f*/*D_f_*. This represents the transmission rate per capita to an average *T_f_* members of the susceptible population per capita by a number *f* of contaminated objects per capita for a duration *D_f_* that viruses survive on a contaminated object.^1^ The form corresponds to an exponential decay in infectiousness of the fomites, where *D_f_* is the mean duration. The epidemic is initiated by the introduction of exposed and infectious carriers at the respective rates *c_e_* and *c_i_* per capita (of the initial population).

The model equations are1
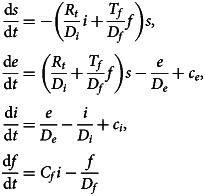
The susceptible, exposed and infectious fractions depend only on the product *N_f_* = *C_f_T_f_*, the number of infection-inducing fomites introduced into the population per day per infectious person.^2^ Initially, *R_t_* = *R*_0_, where *R*_0_ is the basic reproduction number when the epidemic starts.

### Input parameter values

The parameter ranges considered are summarised in Table 1. The estimates for values of the SEIR parameter are taken from Davies *et al*. [9] and Flaxman *et al*. [3] for COVID-19 in the UK. Estimates for the mean duration *D_f_* of SARS-CoV-2 on materials are 0.41 (0.34–0.49 95% CI) day on plastic, 0.34 (0.28–0.41 95% CI) day on stainless steel and 0.21 (0.14–0.30 95% CI) day on cardboard [5], although it is noted that the measurements were under ideal laboratory conditions and may not be applicable in a real-world setting.

**Table 1.** Model parameters

Parameter	Description	Value	Reference
*R*_0_	Initial reproduction number	1.5 < *R*_0_ < 5.5	9, 3
*R*_ld_	Post-lockdown reproduction number	0.3 ≤ *R*_ld_ ≤ 2	3
*R*_lde_	Post-easing reproduction number	0 ≤ *R*_lde_ ≤ 2	Assumed
*N_f_*	Fomite transmission rate (per day per infected person)	0 ≤ *N_f_* ≤ 0.05	Assumed
*D_e_*	Duration of exposed period	4 days	9
*D_i_*	Duration of infectious period	5 days	9
*D_f_*	Duration of fomite infectious period	0.21, 0.34, 0.41 day	5
*c*_0_	Peak source rate per capita	10^−6^ day^−1^	12
*t_c_*_0_	Time of source peak	day 77	12
FWHM	Source distribution FWHM	8 days	12

The number of cases of COVID-19 introduced in the UK is unknown, but estimates suggest at least 1356 infected individuals entered the UK, and likely more, peaking in mid-March (day 77 in the year) at the rate of just under 70 per day with a full-width at half-maximum (FWHM) of about 8 days [12]. A normal distribution with this FWHM fails to capture the tails in the distribution. The source distribution is modelled instead as *c*(*t*) = *c*_0_/[1 + 4(*t*−*t_c_*_0_)^2^/ FWHM^2^], and apportioned to the exposed and infectious carrier sources in proportion to the duration of their respective periods: *c_e_* = *D_e_ c*/ (*D_e_* + *D_i_*), *c_i_* = *D_i_ c*/ (*D_e_* + *D_i_*). Once normalised to the initial rise in death rates, the results after lockdown are found insensitive to these choices.

Although *R_t_* will not have changed to a new fixed value instantaneously after lockdown, for simplicity, lockdown conditions are modelled by taking *R_t_* = *R*_0_ before the lockdown and *R*_ld_ after. After lockdown easing, the reproduction number is taken to be *R*_lde_.

### Means for estimating transmission rates

The posterior parameter values and predicted death rates are based on a maximum-likelihood analysis, where the likelihood of a given model is given by the product of the Poisson probabilities of the reported weekly deaths compared with the mean weekly death rates predicted by the model. The intervals for the modelled parameters listed in Table 1 are sampled uniformly. The derived confidence intervals for a given parameter are given by marginalising the model likelihoods over the remaining parameters to obtain posterior distributions for each parameter.

A mean infected fatality ratio 0.0050 is adopted. This is based on the age-stratified case fatality ratio, adjusted for underestimates from limited case reporting [9], the projected age distribution in the UK for 2020 from the Office for National Statistics [13], and allowing for a factor two smaller infected fatality ratio compared with case fatality ratio [14], as summarised in Table 2. The daily death rate per capita for all cases is estimated from2
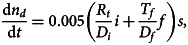
where *n_d_* is the total number of deaths per capita, and allowing for a mean three-week delay from exposure to death [9]. The delay is slightly enlarged to four weeks during the initial rise to ensure the peak death rate is captured, necessary to provide representative infection rates leading into the post-lockdown period. All models assume the same value for *R*_0_ before lockdown to provide a fair comparison.

**Table 2.** Age-stratified case fatality rates from COVID-19 in UK

Age range (years)	Population fraction (ONS projection for 2020)	Case fatality rate (from [9])
0–9	0.12	0.00%
10–19	0.11	0.09%
20–29	0.13	0.10%
30–39	0.13	0.12%
40–49	0.13	0.23%
50–59	0.14	0.68%
60–69	0.11	1.87%
70–79	0.086	4.14%
80–89	0.042	7.68%

By mid-July, it was becoming apparent that the decrease in the incidence rate of COVID-19 in the general population in the UK had levelled off, but was on the rise again in August and September [2]. Rather than model the immediate impact of the initial lockdown and the rise in August and later, only data from weeks 18 to 34 (allowing for a mean three-week delay from onset to death) are used to solve for *N_f,_ R*_ld_ and *R*_lde_. The data used are provided in Table 3.

**Table 3.** Weekly registered deaths in the UK^a^

Week	Registered deaths	Week	Registered deaths
11	5	23	1697
12	114	24	1204
13	607	25	849
14	3801	26	651
15	6888	27	561
16	9495	28	388
17	9008	29	303
18	6680	30	231
19	4426	31	201
20	4214	32	162
21	2872	33	146
22	2000	34	149

a Data reported by the Office for National Statistics [1].

## Results

### Fit parameters

The rise in the number of weekly deaths before lockdown corresponds to *R*_0_ = 3.072 ± 0.003 (95% CL) for the maximum-likelihood model, allowing for uniform sampling over 1.5 < *R*_0_ < 5.5. This is consistent with the range *R*_0_ = 2.68 ± 0.57 estimated by Davies *et al*. [9] from a meta-analysis of published studies.^3^ The results below for indirect transmission are based on the post-lockdown rates, with models assuming 0 ⩽ *N_f_* < 0.05, sampled uniformly over this interval.

The reproduction numbers and infection-inducing fomite rates found for fomite decay times of *D_f_* = 0.21, 0.34 and 0.41 day are summarised in Table 4. They vary little for different values of *D_f_*, as the decay times are very short compared with the evolutionary timescale of the epidemic. They all represent the data equally well. A weighted average of all three (allowing for small differences in variances and likelihoods) after lockdown gives *R*_ld_ = 0.79 ± 0.01 (95% CI) and *N_f_* = 0.009 ± 0.001 (95% CI). The post-lockdown value of *R_t_* < 1 reflects the reduction in the infection rate following lockdown [2, 3].

**Table 4.** Model results.^a^

*D_f_* (day)	*R*_ld_	*R*_lde_	*N*_f_ (fomites day^−1^ (inf. person)^−1^)	*N*_f_f*max/*D*_f_ (infections day^−1^ (susc. person)^−1^)	Relative likelihood
0.21	0.786 ± 0.009	0.994 ± 0.034	0.0086 ± 0.013	(2.3 ± 0.2) × 10^−4^	1.00
0.34	0.785 ± 0.009	0.991 ± 0.034	0.0089 ± 0.013	(2.4 ± 0.2) × 10^−4^	1.02
0.41	0.784 ± 0.009	0.991 ± 0.034	0.0090 ± 0.013	(2.4 ± 0.2) × 10^−4^	1.01
Avg	0.785 ± 0.009	0.992 ± 0.034	0.0088 ± 0.014	(2.4 ± 0.2) × 10^−4^	
−	0.842 ± 0.003	0.922 ± 0.032	0	−	

a Indicated uncertainties show 95% CI. The ‘Avg’ in the fourth row is the statistical average over the cases *D*_f_ = 0.21, 0.34 and 0.41 day. The last row with *N*_f_ = 0 corresponds to the case with no fomites. The second to last column shows the peak rate of infections from fomites per susceptible person per day.

The UK began to ease the lockdown on 4 July 2020. The decline in the fraction of the population in England testing positive for COVID-19 levelled off over the following week [2]. The average reproduction number found from a maximum-likelihood fit to the numbers of registered weekly deaths after easing is *R*_lde_ = 0.99 ± 0.03 (95% CI). Significantly, a value exceeding unity is included in this range, suggesting the epidemic may have already returned to a growing phase by August.

**Fig. 1.**
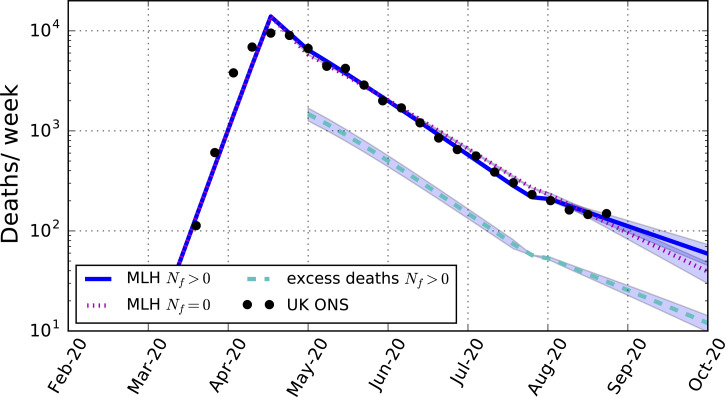
Predicted weekly death rates for the maximum-likelihood (MLH) model including fomite transmission (blue solid line, with 95% CI), excess deaths compared with a counterfactual model assuming the same reproduction numbers as for the maximum-likelihood model but without fomite transmission (cyan dashed-line, with 95% CI), and the maximum-likelihood model assuming no fomite transmission (magenta dotted-line, with 95% CI). The data points are the total weekly number of deaths in the UK due to COVID-19 as reported by the Office for National Statistics (Table 3).

Compared with a counterfactual model with the same values of *R*_ld_ and *R*_lde_ as for the best-fitting model with fomites, the model including fomites suggests the presence of fomites contributed to an increase in the total number of deaths by about 25%, as shown in Figure 1 (dashed cyan line). These arise both through contamination by fomites and the subsequent direct transmission by the consequent infectious cases to the susceptible population.

### Illustrative case: postal deliveries in UK

To give the constraint on *N_f_* some context, potential indirect transmission by delivered post in the UK is considered. The Royal Mail adheres to public health guidelines for its employees, and it has placed several further protective measures in place in the delivery of post to customers [15]. Potential points of further accidental contamination not readily eliminated are the distribution of post to post carriers and during the sorting and final delivery to customers.

**Fig. 2.**
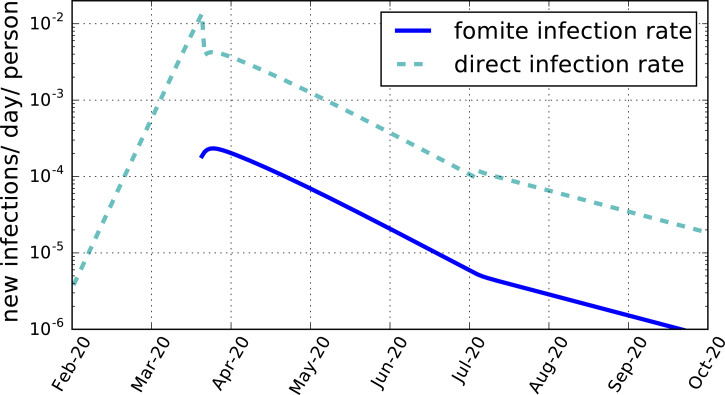
The average daily rate of new infections per susceptible person per day produced by fomites, *T_f_ f*/*D_f_* (blue solid line), and by direct transmission, *R_t_ i*/*D_i_* (cyan dashed line), both for the maximum-likelihood model for a mean fomite duration time *D_f_* = 0.2 day and *C_f_* = 0.57 day^−1^ capita^−1^.

Approximately 14 billion letters and parcels are delivered per year by the Royal Mail [16]. The number of objects delivered per day per capita for a UK population of 67 million is then *C_f_* = 0.57 day^−1^ capita^−1^.^4^ The lifetime for SARS-CoV-2 on post is unknown. The value *D_f_* = 0.2 day for cardboard is adopted. The maximum-likelihood model for *N_f_* ⩾0 gives *T_f_* = 0.015 ± 0.002 (95% CI). Thus, only an average of three in 200 contaminated articles transmits the illness. Since other fomites may be expected to be present, this should be regarded as an upper limit, *T_f_*  < 0.017 (98% CI). The corresponding transmission rate is shown in Figure 2. At its post-lockdown peak, the transmission rate by fomites is about 2 × 10^−4^ per day per susceptible person (Table 4). By the end of the lockdown period, it has declined to under 5 × 10^−6^. These are well below the direct transmission rates of about 4 × 10^−3^ per day per susceptible person at its post-lockdown peak, and 10^−4^ at the end of lockdown. None the less, the slowing down by fomite transmission of the reduction in the total infection rate during the lockdown may have been sufficient to increase the death rate by as much as 25% (Fig. 1).

## Discussion

### Effect of fomites on epidemic evolution

Because of the practical difficulties involved in making direct measurements of the transmission rate of COVID-19 through fomites, a global population approach is adopted. It is found that adding a fomite term to the standard SEIR equations greatly improves the agreement of the model with the weekly death rate from COVID-19 reported in the UK.

Compared with a best-fitting model with no fomites (*N_f_* = 0), shown in Figure 1, with post-lockdown reproduction number *R*_ld_ = 0.84 (Table 4), a somewhat smaller reproduction number value (*R*_ld_ = 0.79) is required to match the data when fomites are allowed for. The lower reproduction number is compensated for by the additional contributions from fomite transmission.

A less intuitive consequence of fomite transmission is the larger reproduction number after lockdown is eased when allowing for fomites, *R*_lde_ = 0.99, compared with the fit with no fomites, *R*_lde_ = 0.92, a value that the fit including fomites excludes with over 99.9% confidence. The value for the fit without fomites is smaller because the infection rate was declining less slowly in the model before lockdown was eased compared with the model including fomites, as shown in Figure 1. To match the relatively small death rates after the lockdown was eased requires a smaller reproduction number than the model allowing for fomites. This shows that not allowing for fomites in a model may lead to an under-estimate of the reproduction number following a reduction phase in the epidemic. In the case modelled, the reproduction number found in the model with fomites includes within its 95% confidence interval *R*_lde_ > 1, so that the epidemic in the UK may have already re-entered a growing phase by August.

Direct verification of a fomite contribution would help validate the model, but this is made difficult by the low prevalence of infectious-inducing fomites, as shown in Figure 2 and Table 4. The most direct means of ascertaining the contribution of indirect transmission may be through direct random testing for contaminated material. As illustrated for UK postal deliveries, however, at most only a few in a thousand letters and parcels delivered in a day would be contaminated. Post-lockdown easing, the numbers are even smaller, below one in 10,000. This would require the testing of tens of thousands of independent, randomly selected delivered articles, which is likely prohibitive. Another approach would be to search for a statistically significant increase in COVID-19 among recipients of post from infectious (pre-symptomatic) postal workers later verified by testing to have been ill, but the numbers again will be small.

Studies similar to this one could be repeated for other countries to see if similar improvements in matching the data are found, particularly if similar values of *N_f_* were found. Smaller, isolated environments may also be modelled, although small samples are increasingly prone to variations particular to each case. Cruise ships [18, 19], and possibly large work spaces [20], may be especially helpful for establishing the production rate and prevalence of fomites. Surveys of potential fomites even in non-infected environments would help to assess how frequently fomites may be introduced into a given environment that could provide data for epidemic population modelling.

### Limitations

Further measurements of the duration of SARS-CoV-2 on substances in real-world situations are required. Other factors than direct transmission and fomites may also contribute to the spread of the illness, such as aerosols, blood, urine and faeces, although transmission by any of these has not been demonstrated conclusively [4]. The differences found here from a model allowing only for direct transmission may partly, or even entirely, arise from other means of transmission such as these. Alternatively, it could reflect a continuously evolving reproduction number *R*_t_. The relative simplicity with which the fomite term improves the fit to the data, however, would seem to argue in its favour.

Both direct and indirect transmission rates may differ among sub-populations of different ages. Allowing for age-dependent transmission rates and transmission between age groups would further add to the uncertainty in the contribution by fomites. Another limitation of the SEIR model is that it implicitly assumes exponential distributions for the exposed and infectious phases. The actual distributions are still unknown [21]. Other statistical distributions may prove more accurate once more data become available.

A maximum-likelihood approach requires a probabilistic model for the data. In this study the weekly reports of the number of registered deaths in the UK resulting from COVID-19, as reported by the Office of National Statistics, were used. The numbers were modelled by the minimal assumption of Poisson fluctuations, as these depend only on the reported numbers. The determinations are based on a combination of testing and physician assessments. As such they are prone to testing limitations and possibly subjective judgement. Large day-to-day variations are found, suggestive of large correlations in time. Following ONS practice, weekly numbers were used to smooth these fluctuations and suppress their correlations. Further understanding of the nature of the fluctuations and possible remaining week-to-week correlations would likely broaden the error estimates provided here. These uncertainties are common to any population models of the epidemic.

### Policy implications

The possibility of transmission from fomites may be especially relevant to policies designed to protect the more than two million clinically extremely vulnerable people in the UK, as self-shielding alone may not be adequate. Modelling differences in the infection rates between shielded and unshielded sub-populations may be a means of determining how great a risk factor indirection transmission is. If the risk of indirect transmission through postal deliveries is assessed to be a significant contributor to the spread of COVID-19, a possible means of mitigation is the effective use of face coverings, under appropriate guidance [22], by postal workers coming into direct contact with postal items within a day of delivery. A solution considered in the context of re-using PPE equipment is heating used equipment or exposing it to UV radiation [23]. Such an approach could be considered for post, such as exposure to sunlight for periods of several minutes to a half hour [24], and for other articles that commonly come in contact with the public such as food packages. The tests on PPE equipment, however, were inconclusive in terms of required dosages in realistic scenarios [23]. It is unknown how effective exposure to sunlight would be on post in a realistic environment; post is also often concealed until delivered for security reasons, so procedural adjustments would be required. Until improved assessments are made, or other means of removing or preventing contamination become available, perhaps the simplest advice to give the public is to isolate potentially contaminated articles for 24 h before handling or at least to wash their hands after doing so.

## Conclusions

A maximum-likelihood analysis of a SEIR model with an added fomite term applied to the COVID-19 epidemic in the UK suggests a significant fomite contribution, with 0.009 ± 0.001 (95% CI) infection-inducing fomites introduced into the environment per day per infectious person. The fomite term significantly shifts the inferred values of *R_t_* compared with best-fit non-fomite solutions. It is suggested that fomites be incorporated into more refined stochastic models and simulations to better assess the effectiveness of non-pharmaceutical interventions in curbing the epidemic.

## Data availability statement

All the data used to support this study are available through the cited references.

## Footnotes

1 For simplicity, an article that comes into close proximity to an infectious carrier is considered contaminated, and the average effectiveness of the contaminated article to transmit the illness is quantified through *T_f_*.

2 This may be seen by introducing the variable *f** defined by *f* = *C_f_ f*.* Then the first two equations in Eq. (1) become d*s*/d*t* = *-(R_t_ i*/*D_i_ + N_f_ f**/ *D_f_)s* and d*e*/d*t* = *(R_t_ i*/*D_i_ + N_f_ f**/ *D_f_)s*-*e/D_e_ + c_e_*, the final equation becomes d*f**/d*t = i* – *f**/*D_f_* and the third equation remains unchanged. Thus any constraints from infections and their consequences are on *N_f_*, and not the particular kind of fomite, except weakly through *D_f_*. Only actual fomite numbers depend on a particular choice for the value of *C_f_*; the exposed and infectious populations and the consequent fatalities depend instead on *N_f_*.

3 Using the determination of *R*_0_ from [9] as a prior makes little difference to the derived parameter values once *R*_0_ < 1.5 is excluded.

4 The delivery rate is assumed to differ little from the mean for 2018−2019. Whilst the volume of letters delivered fell by 33% from April to May 2020, the volume of parcels increased by 37%. For the full year 2019–2020, there was little difference in the net volume of delivered letters and parcels from the previous year [17].
